# Exploiting Matrix Stiffness to Overcome Drug Resistance

**DOI:** 10.1021/acsbiomaterials.4c00445

**Published:** 2024-07-05

**Authors:** Hakan Berk Aydin, Altug Ozcelikkale, Ahmet Acar

**Affiliations:** † Department of Biological Sciences, Middle East Technical University, 06800, Ankara, Turkey; ‡ Department of Mechanical Engineering, Middle East Technical University, 06800, Ankara, Turkey; § Graduate Program of Biomedical Engineering, Middle East Technical University, 06800, Ankara, Turkey

**Keywords:** cancer drug resistance, extracellular matrix, matrix stiffness, tumor microenvironment, matrix biology

## Abstract

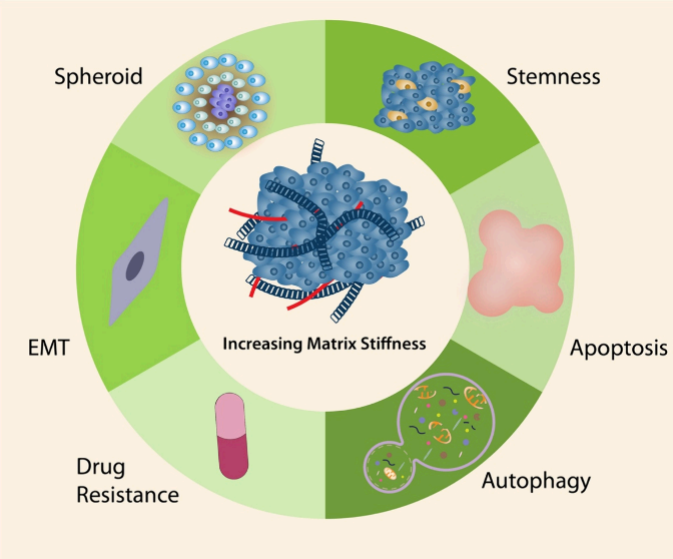

Drug resistance is arguably one of the biggest challenges facing cancer research today. Understanding the underlying mechanisms of drug resistance in tumor progression and metastasis are essential in developing better treatment modalities. Given the matrix stiffness affecting the mechanotransduction capabilities of cancer cells, characterization of the related signal transduction pathways can provide a better understanding for developing novel therapeutic strategies. In this review, we aimed to summarize the recent advancements in tumor matrix biology in parallel to therapeutic approaches targeting matrix stiffness and its consequences in cellular processes in tumor progression and metastasis. The cellular processes governed by signal transduction pathways and their aberrant activation may result in activating the epithelial-to-mesenchymal transition, cancer stemness, and autophagy, which can be attributed to drug resistance. Developing therapeutic strategies to target these cellular processes in cancer biology will offer novel therapeutic approaches to tailor better personalized treatment modalities for clinical studies.

## Introduction

Cancer remains one of the major global health problems. The numbers have scaled up to 20 million diagnosed and nearly 10 million deaths from cancer globally.^1^ Estimations for new diagnoses and death in 2024 are around 2 million and 611,000, respectively.^2,3^ These statistics show that cancer remains to be a leading cause of death worldwide despite that significant efforts to understand the disease and extraordinary progress in its treatment have been in place. Tumor biology is complex to study, partly owing to the genetic and phenotypic variations across cancer cell populations within the tumor tissue, different tumor sites, and patient-to-patient heterogeneity.^4^ Tumor heterogeneity significantly affects disease prognosis, including response to chemotherapy or other treatment modalities.^5,6^ One of the contributing factors to this problem is the complexity of the microenvironment, where heterotypic cells are embedded in the extracellular matrix (ECM) with a unique composition that varies from tissue to tissue.^7,8^ The tumor microenvironment (TME) is the combination of tumor’s dynamic interactions between the signaling molecules secreted by fibroblasts, blood vessels, immune cells, which significantly impact cell growth, migration, differentiation, and survival via aberrant activities of signal transduction pathways.^7,9−11^ Efforts toward in vitro and in vivo characterization of the TME over the past few decades have led to a significant body of multidisciplinary research that improved our understanding of the role of the microenvironment on tumor progression and metastasis.^12,13^ One of the primary outcomes of this research has been the recognition of the mechanical properties of the TME as an important microenvironmental cue guiding cancer cell biology.^14^ The ECM remodeling takes place while these changes occur. One of the outcomes of the ECM remodeling is the alteration of the stiffness of the matrix. This can cause several changes in the dynamic nature of the ECM and TME. Since the stiffness of ECM is primarily based on the cross-linking density of the ECM, matrix stiffness studies mainly focused on the changes of ECM with increased stiffness.^15^ The studies led to the recognition that matrix stiffness directly or indirectly affecting fundamental cellular processes such as tumor initiation and tumor growth resulting in proliferation, hyperplasia, dysplasia, and migration.^16−18^ In the light of these findings, one research branch started to focus on a mechanistic understanding of the effects of TME on drug resistance which can occur either as the disease progresses or in response to therapies.^19−21^

The duration and cost of the drug discovery are estimated to take more than 10 years and more than 2 billion dollars, respectively,^22^ with a failing rate of 90% until a drug is approved by the FDA.^23^ The failure of drug development programs has several reasons including mainly due to the lack of clinical efficacy.^23^ The probability of the launch of the cancer drugs is the least especially in phase III trials.^24^ The drug classification for cancer treatment can be divided into, chemotherapy, hormonal therapy, immunotherapy, and targeted therapies. Therapeutic agents used in the treatment of cancer patients can be subclassified according to their mechanism of action.^25^ For example, alkylating agents, namely cisplatin, and oxaliplatin, target proteins and nucleic acids to inhibit DNA replication or transcription. Furthermore, antimetabolites such as fluorouracil, cytarabine, methotrexate, and azacitidine can inhibit DNA replication. Another class is the antimicrotubular agents such as doxorubicin, irinotecan, paclitaxel, docetaxel, and vinblastine, which target mainly topoisomerases.^26−31^ A list of subclasses of chemotherapeutic agents is presented (Table 1).

**Table 1:** Classes of Commonly Used Chemotherapeutics with Their Mechanisms of Action and Their Chemoresistance Mechanisms

Drug Class	Drug	Mechanism of Action	Chemoresistance Mechanism	ref
Antimetabolites	5-Fluorouracil	Inhibition of thymidylate synthase (TS)	Drug efflux	(48)
Antimetabolites	Gemcitabine	Inhibition of DNA synthesis	EMT, Inflammation	(49−51)
Antimetabolites	Methotrexate	Inhibition of dihydrofolate reductase (DHFR)	Reduced uptake, drug efflux	(52,53)
Alkylating Agents	Cisplatin	Interfering with DNA replication	Drug efflux, autophagy, reduced uptake, CSC	(54−57)
Alkylating Agents	Oxaliplatin	Inhibit DNA replication	Reduced uptake, drug efflux, autophagy	(58,59)
Topoisomerase Inhibitors	Doxorubicin	Disruption of DNA repair	Drug efflux, apoptosis inhibition, MAPK/ERK	(60,61)
Topoisomerase Inhibitors	Irinotecan	Inhibiting the topoisomerase I	Drug inactivation, drug efflux	(62,63)
Mitotic Inhibitors	Docetaxel	Inhibition of microtubule depolymerization	Drug influx/efflux, CSC	(64−67)
Mitotic Inhibitors	Paclitaxel	Interfering with tubulin to block G2/M phase of cells	Drug efflux, subsequent apoptosis, autophagy	(68−71)

**Figure 1:**
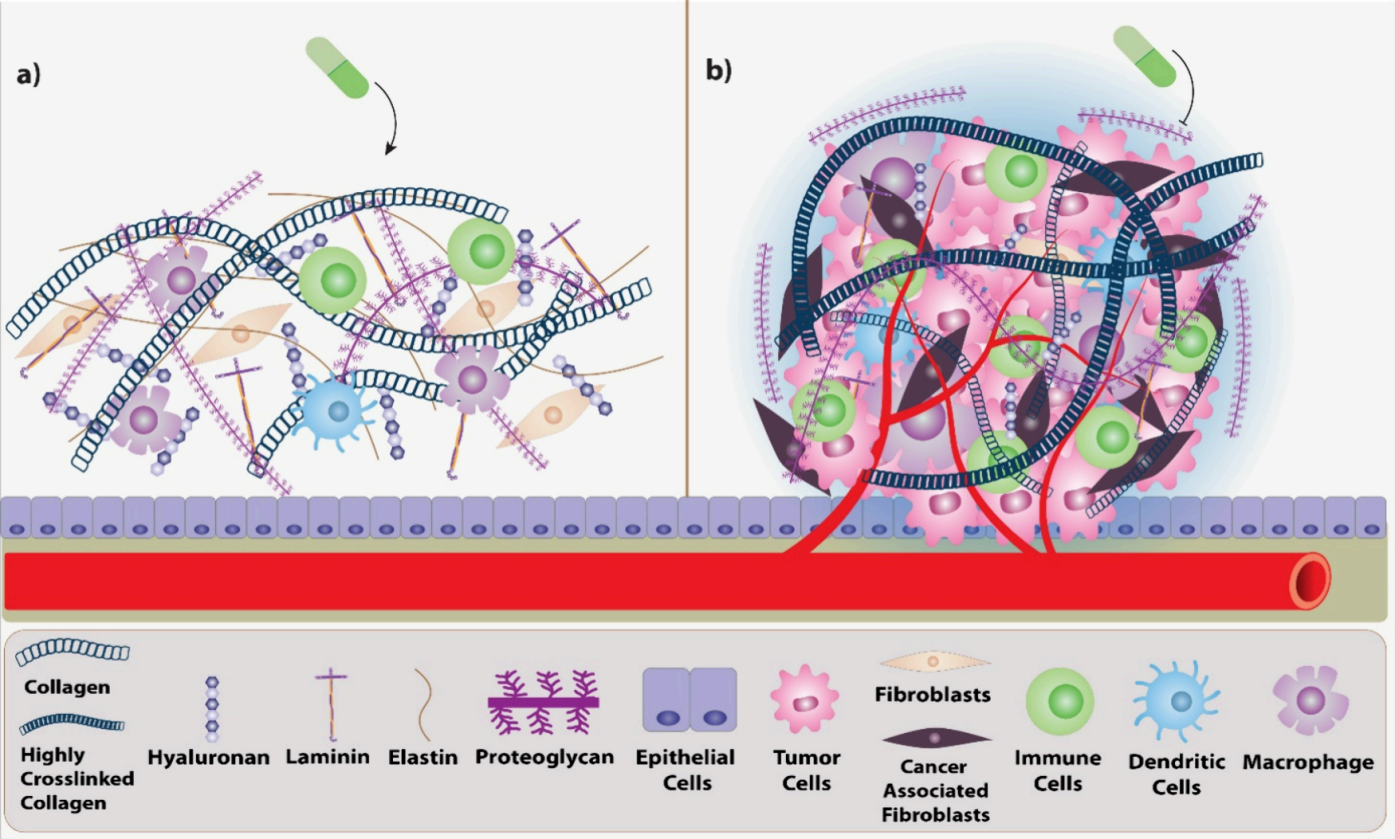
Schematic representation of the relationship between the normal extracellular matrix (a) and tumor microenvironment (b). Matrix deposition and cross-linking levels are closely related to the matrix stiffness in the tumor microenvironment alongside with the immune cell types and cancer associated fibroblasts. Various signaling pathways, such as Hippo/YAP1, Notch, Wnt, YAP/TAZ, TGFβ, PI3K/AKT/mTOR, MAPK, ERK, and JAK, are involved in drug resistance and cellular processes, including tumor growth, EMT, cancer stemness and autophagy.

Drug resistance in cancer is one of the leading major reasons for the treatment failure seen in cancer patients, and this impacts the survival rate.^32^ Different mechanisms involved in cancer drug resistance have been proposed including the genetic factors, and nongenetic factors.^33^ The nongenetic factors include an activation of the crosstalk between different signaling pathways, phenotype switching, and increase/decrease of drug uptake or efflux.^34^ Another possibility of overcoming low percentages of drug success in the preclinical and clinical studies, next-generation technologies, such as organ-on-a-chip systems and synthetic or hybrid hydrogels and their interactions with 3D cell culture systems, such as organoids, spheroids or tumoroids can be implemented into the preclinical stages of drug development.^35^ Recent advances in tissue engineering and biomimetic approaches have accelerated the development of preclinical drug design and screening systems to understand the mechanisms of drug resistance toward their better use in personalized medicine.^36^ Furthermore, the recognition of new technologies by regulatory bodies such as the recent FDA Modernization Act 2.0 in the U.S.,^37^ as well as European Union’s several regulatory actions along with individual countries such as Germany, Netherlands, Italy, Switzerland and United Kingdom,^38^ shows that mimicking normal ECM and its change into TME have become important. Through mimicking the TME, cancer progression can be studied including the natural biomaterials such as collagen or fully synthetic polymers or bioconjugated synthetic polymers.^39−44^ Mimicking the TME has been achieved in several ways including the mechanobiological approaches, which focuses on the mechanical properties of the TME, and ECM, and their effect on the cell, tissue, or signaling pathways, and the utilization of genetically engineered animal models.^45^ Overall, these efforts and approaches have helped to gain a better understanding of underlying mechanisms of drug resistance and to ultimately develop strategies to overcome this very problem. The normal ECM and TME and their changes in drug resistance based on mechanobiological approaches are summarized (Figure 1).

Among the mechanical cues presented by TME, the matrix stiffness comes forward as a critical influencing factor for growth, progression, transformation, invasion, and metastasis. Stiffness can be defined as the material’s inherent resistance to deformation under specific loading conditions, encapsulating a spectrum of mechanical responses to external forces including tensile, compressive, shear, or torsional strain as a result of internal stresses that develop in the material. Another important factor for the relationship between TME and solid tumor is the abnormalities in biomechanical factors in TME which can disrupt the interstitial fluid pressure (IFP) of the organs. This can either be done by hyperpermeable blood vessels or compression of blood vessels by solid stress. Increasing IFP can result in angiogenesis, fibroblast activation, induction of MMPs, and metastasis. Also, via the addition of integrin focused mechanotransduction, it can be related to Notch, TGFβ, and YAP/TAZ.^141^ Characterizing stiffness involves employing diverse techniques, with stiffness measurements expressed in terms of different moduli, each associated with various factors including specific material models, loading conditions, and length scale of measurements.^46,47^ Hence, a nuanced understanding of stiffness measurement as outlined in this review is imperative for accurate comparison and interpretation of findings for mechanobiological effects of stiffness across scientific literature.

This review aims to summarize the recent advances in the targeting of TME with a particular focus on the effect of matrix stiffness on various cellular processes involved in tumor progression and metastasis to shed a light on signal transduction pathways facilitating epithelial-mesenchymal transition, cancer stemness and autophagy involved in cancer drug resistance.

## Extracellular Matrix

### Composition and Structure

The most significant part of the TME is that the ECM, cells and their environment are highly dynamic, and they can quickly alter their mechanical properties to respond or adapt to specific changes.^72,73^ They can adapt different cellular responses between cell–cell interactions and cell–ECM interactions.^74−76^ Cancer cells interact with the ECM primarily to form defined tissue structures.^10,77^ The components of ECM can be a part of various structural and elastic dynamics.^78^ The ECM is a porous biopolymer network that is composed of fibrous proteins such as collagens, elastins, fibronectins, and laminins as well as a family of proteoglycans and glycosaminoglycans bound to the protein core and a unique nonsulfated glycosaminoglycan, hyaluronic acid^79^ where the pores are with physiological fluids. The dynamic nature of ECM is related to changes in their glycosaminoglycan composition and therefore the viscoelasticity.^80^

Fibrous proteins are responsible for mediating elasticity and tensile strength by regulating cell adhesion and tissue development. Collagen is the most abundant fibrous protein in the ECM with more than 30% of all proteins. Most collagen types have three alpha helical coils that are soluble in water.^7,81^ There are three main types of collagens which are fibrillar, fibril-associated, and nonfibril collagens.^82^ The fibrillar collagen composition of the ECM is critical for the structural changes of the tissue. The increasing collagen levels in the tissues promote tumor invasiveness and progression.^83−85^ The degradation of collagen is caused by the family of a protease family, matrix metalloproteinases (MMPs).^86,87^

Elastin is another fibril protein which is not soluble and is found broadly as cross-linked by tropoelastin, a water-soluble protein, via lysyl oxidase (LOX) and is highly associated with tissue recoil after stretching due to its dynamic 3D structure.^88,89^ The damage in elastin will increase the elastin-like and elastin-derived peptide synthesis and is known to increase the tumor growth.^90^ Fibronectins are critical for the mechanoregulation with the presence of the arginine-glycine-asparagine (RGD) sequence facilitating the binding of cells to adhesion molecules such as integrins.^91^ This process mediates cell growth and differentiation and has a role in angiogenesis, tumor progression, and metastasis.^92−94^ Several growth factors such as fibroblast growth factor (FGF), insulin-like growth factor (IGF), transforming growth factor β (TGF-β), platelet-derived growth factor (PDGF), and hepatocyte growth factor (HGF) are known to interact with fibronectins.^80,95^ The increasing level of fibronectin, especially in ECM or basement membrane (BM), is observed in malignant tumors which is primarily caused by the upregulation of several signaling pathways such as focal adhesion kinase (FAK), phosphatidylinositol-3-kinase (PI3K)/AKT, and extracellular signal-regulated protein kinase (ERK1/2).^96^

Laminin is not a fibrous protein but is considered as a glycoprotein and it is one of the most abundant proteins after collagen in ECM and BM.^80,81,97^ Interaction with different ECM components, such as collagen or fibronectin causes the laminin to regulate cell adhesion, migration, morphogenesis, and tissue homeostasis.^98^ Proteoglycans have a protein core and are covalently bonded with glycosaminoglycans.^99^ Hyaluronic acid does not contain any protein core; hyaluronic acids have a linear polysaccharide, hyaluronan, in their core.^7,79^ Proteoglycans are mainly in charge of hydration and compressive strength, which are directly related to the elastoviscosity of the ECM.^9,79^ Recent studies show that changes in hyaluronic acid levels in serum can be considered as a biomarker of breast cancer since the changes in the hyaluronic acid composition are associated with tumor progression.^100^

### ECM Mechanics and Matrix Stiffness

The ECM can proportionally reach a dynamic balance via cells’ secretion of proteins and signaling molecules as well as the cross-linking of several proteins. The cross-linking of the ECM can be achieved in several ways, one of which is lysyl oxidases.^101,102^ These molecules can be secreted by cancer-associated fibroblasts (CAFs) and cross-links the collagen fibrils and elastins covalently.^103^ The overexpression of LOX family enzymes can induce invasiveness, metastasis and desmoplasia via increasing the stiffness of the ECM.^104^ A study by Rossow et al.^105^ showed that a LOX-mediated increase in collagen expression and cross-linking can could cause doxorubicin resistance in different cancer cell lines. The inhibition of LOX can result in reversing effect in drug response.^106^ In addition, targeting other matrix cross-linkers such as the LOX family has been shown to be successful in decreasing the matrix stiffness. For example, PXS-5505, a lysyl oxidase inhibitor, used in the treatment of post-polycythemia vera or post-essential thrombocythemia myelofibrosis patients exhibited promising results in decreasing the stiffness in a Phase I/II study.^107^ Targeting LOXL2 has also been effective in softening the matrix stiffness via two different targeted therapies in patients, namely, PAT-1251^108^ and PXS-5382A.^109^

Matrix metalloproteinases (MMPs) are a family of enzymes that can be a part of proteolytical degradation of ECM components.^110^ MMPs can degrade collagen networks in the ECM, which can help soften the matrix.^111^ Overexpression of MMPs can help lowering the already stiffened matrix for overcoming fibrosis and tumorigenesis through ECM breakdown and hence reverting the tumor growth, and angiogenesis.^110,112,113^ Moreover, collagenases, a subgroup of MMP enzyme family, have been reported to cleave the collagen, the most prominent part of stiffed matrix, and therefore reducing the matrix stiffness.^114^

When tissue mechanical properties are considered, different components of tissues should be addressed. Mechanical properties govern the degree of deformation the tissue undergoes under a given loading condition.^115^ These material properties can be classified as isotropic and anisotropic depending on whether they are independent of or dependent on the direction of characterization.^116^ Tissue mechanical properties are largely anisotropic, rendering the direction of stress highly important, especially for defining viscoelasticity of tissues.^117,118^ The stiffening matrix can cause several types of stress to the tissue, including mechanical stress. The stress and compression on a cell affect the cells adapting their environment dynamically and modifying its microenvironment. These effects can change cell proliferation, plasticity, enhancing stem cell characteristics, inducing autophagy, and increase the therapeutic response.^14,119−123^ Since there is no strict definition for stiff or soft, the stiffness of material is not absolute. For example, the softest tissue can be considered mucus^124^ in the human body, and bone is the most rigid tissue.^125^

The stiffness of a TME is an emerging research area since much recent literature shows that the stiffness of a microenvironment is directly related to the hallmarks of cancer.^87^ Also, the stiffness of a tissue is highly associated with cell adhesion molecules (CAMs) (e.g., integrins, FAK) and several signaling pathways (e.g., YAP/TAZ, Rho/ROCK, MAPK, etc.).^126−129^ These signaling pathways can be induced directly or indirectly by the ECM remodeling and the matrix stiffness.

Since the stiffness affects the function of a cell directly, the stiffness of TME and the living tissues plays a critical role ranging from tissue engineering to cancer research. Studies show that the increased tissue stiffness is highly characteristic for solid tumors in breast, colorectal, or pancreatic cancers.^130,131^ TME stiffness can be seen via various origins, and the cancer-associated fibroblasts (CAFs) play a highly active role in tumor fibrosis for most cancer types.^132^ CAFs also play an essential role in regulating biophysical and biomechanical properties of tumors by causing compressive stress and the proliferation of epithelial cancer cells.^133,134^ In the study of Xiao et al., they prepared a 3D coculture system with CAF and PDAC organoids in commercially available Matrigel with increasing level of Collagen-I. The stiffer matrix promoted YAP1 intensity in CAFs more than softer ones. They also showed that CAFs stiffen the environment through a LOX based cross-link. And the exosome level increase related to drug resistance, but inhibiting exosomes, can decrease the stiffness associated with drug resistance.^135^ Also, CAFs can promote the epithelial to mesenchymal transformation (EMT) and neoangiogenesis, new blood vessel formation, which exhibits an essential role in cancer metastasis.^136^ The stiffness is directly correlated with the progression of cancer in vivo.^137^ The change of biophysical activity in ECM affects TGF-β activation.^138^ The strained ECM will help the conformational change of latency-associated peptide (LAP) and release the TGF-β1.^139^ Tumor-Associated Collagen Signature-3 (TACS-3) causes an increase in the stiffness and loss of elasticity in the ECM, especially in ovarian cancers.^98,140^

### Stiffness Characterization

Characterizing stiffness involves employing diverse techniques, with stiffness measurements expressed in terms of different moduli, each associated with various factors including specific material models, loading conditions, and length scale of measurements.^46,47^ For example, Young’s modulus, determined through uniaxial tension or compression testing, quantifies the material’s length change under extension or compression. Similarly, the dynamic interaction of loss and storage moduli, as observed through techniques like rheometry and dynamic mechanical analysis (DMA), provides insights into viscoelastic behavior and energy dissipation mechanisms within the material.^142,143^ In this manner, the exact meaning of stiffness as an umbrella term and the diverse set of stiffness measurements reported in the literature depend upon the specifics of the characterization approach with features and limitations that need to be understood for proper interpretation of findings in the literature. In this section, we outline basic features of common stiffness characterization techniques employed in matrix stiffness-related studies.

**Figure 2:**
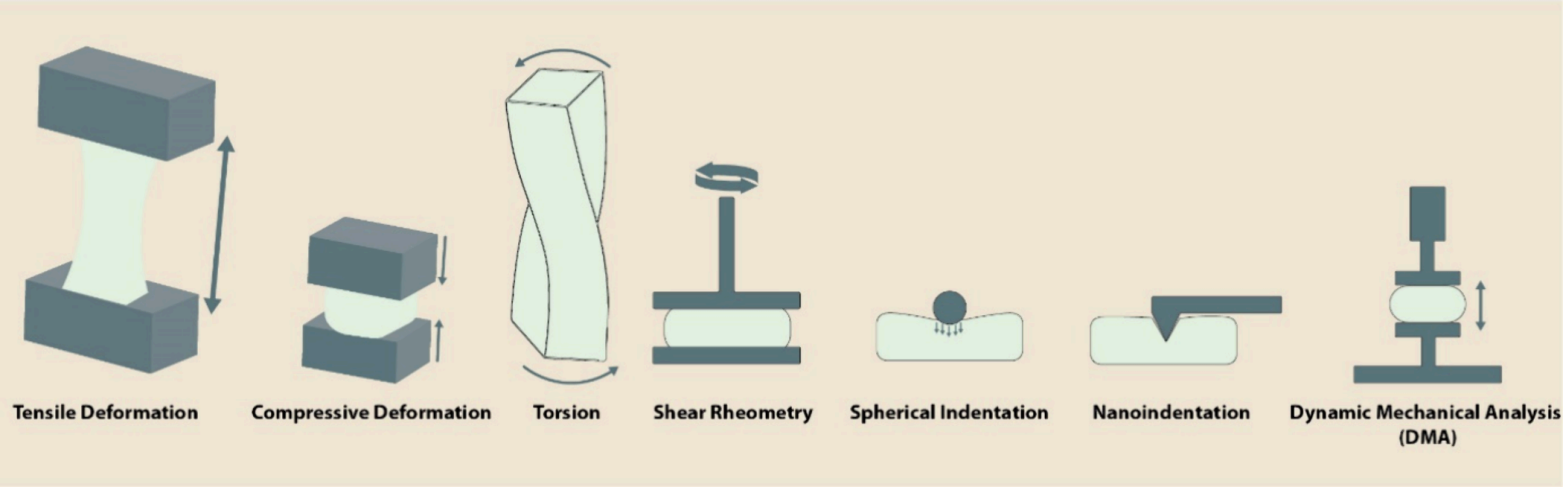
Mechanical characterizations are linked to mechanical deformation systems. Tensile and strain deformations can result in static deformations with a complex curve to regulate Young’s modulus while dynamic analysis is based on viscoelastic behavior of the materials to yield storage (elastic deformation) and loss modulus (energy dissipation).

Uniaxial or biaxial tensile testing can be done by applying loading to the tissue along one or two primary directions, respectively. Features of the resulting stress-strain curve, such as the extent and the slope, will determine the tensile strength and elastic modulus of the material under static, or relatively low loading/strain rates. In a similar approach, using uniaxial compressive loading can be used to determine the compressive elastic modulus.^144,145^ In this point of view, both approaches will quantify the stiffness based on the elastic modulus of the tissue, which can vary significantly under tensile and compressive loading. On the other hand, DMA can be done by applying similar tensile or compressive loading but in a cyclic manner where spring-like elastic and viscous fluid-like characteristics that give rise to energy storage and dissipation in the material can both be quantified effectively.^146,147^ These types of basic mechanical tests mainly characterize bulk tissue properties. On the other hand, Hertzian contact mechanic-based indentation methods are focused on local tissue property characterizations.^148,149^ Indentation can be used for material analysis at micro or nano scale level with very small—on the order of micrometers to nanometers—indentation of the probe to sample. Topographic characterization with nanoindentation can be done by atomic force microscopy (AFM).^150^ AFM can also be used to screen the mechanical properties of the TME, such as changing stiffness in several parts of the TME and the cell itself.^151^ Optical tweezers can be used combined with trapping nanoparticles to characterize soft biomaterials via a range of moduli.^152−154^ X-ray diffraction (XRD) measure the stiffness of ECM components. For example, the measurement of the stiffness of collagen molecules can be done by XRD. Their elastic modulus is between 3 and 9 gigapascals (GPa). The mechanical deformations used in characterization methods are summarized (Figure 2).

For tissue characterizations, optical techniques are also essential for quantifying the changes in the microenvironment. Confocal microscopy can be used to characterize the TME with several different modes, such as reflectance or fluorescence confocal microscopy.^155^ For imaging purposes of TME, nonlinear imaging like multiphoton microscopy and second harmonic generation can be used, especially for live imaging of composition and architecture change of the TME.^156−158^ As a noncontact method, Brillouin microscopy is used to map the stiffness of different biological samples in 2D and 3D. Since Brillouin microscopy is a noninvasive measurement technique, it can potentially be adapted for obtaining in vivo measurements of tissue mechanical properties with subcellular resolution.^159−161^ Other noninvasive methods such as magnetic resonance elastography,^162^ ultrasonography,^163^ and optical coherence tomography^164,165^ are also widely used in measuring the mechanical properties of both healthy normal and cancer tissues in vivo since they are based on elastography. One of the most commonly used stiffness measurement techniques for biomaterials or synthetic materials in biomedical research is shear rheometry. Based on shear stress or shear strain, rheological measurements can range from pascal to megapascal levels.^166−172^ Mechanical characterization techniques and the associated measure(s) of stiffness commonly employed in matrix stiffness literature are presented (Table 2).

**Table 2:** Mechanical Characterization Techniques and the Associated Measure(s) of Stiffness Commonly Employed in Matrix Stiffness Literature

Characterization Technique	Measure of the Stiffness	ref
**Tensile Deformation**	Elastic Modulus	(173)
**Compressive Deformation**	Elastic Modulus	(173, 174)
	Compressive Modulus	(175)
	Storage Modulus	(176)
**Dynamic Mechanical Analysis**	Loss Modulus	(177)
	Storage Modulus	(178)
**Optical Tweezers**	Elastic Modulus	(179)
	Loss Modulus	(154)
	Shear Modulus	(180)
	Storage Modulus	(154)
**Atomic Force Microscopy**	Elastic Modulus	(181−183)
	Shear Modulus	(184, 185)
**Nanoindentation**	Elastic Modulus	(186)
	Loss Modulus	(187, 188)
	Storage Modulus	(187−189)
**Brillouin Microscopy**	Longitudinal Modulus	(190, 191)
**Shear Rheometry**	Elastic Modulus	(168)
	Loss Modulus	(166, 169, 170)
	Shear Modulus	(167, 171, 172)
	Storage Modulus	(192, 193)

The knowledge of tissue stiffness, once properly charterized, can be used towards investigation of cellular tractions. Contractile force transmission between cells and the cell environment created by actomyosin and these cellular forces can be classified as cellular tractions.^194^ Cellular tractions can be measured since they are making shape deformation to materials.^195^ There are many ways to measure these deformations, such as mapping deformation and using synthetic materials with well-known mechanical properties.^196^ In practice, the deformation of the material caused by the cell can be determined by traction force microscopy (TFM) in 2D and 3D.^197,198^ In TFM measurements, polymer hydrogels such as polyacrylamide or polydimethylsiloxane (PDMS) coated with fluorescence nanoparticles as fiducial markers are used to track deformation by cell based on images from wide-field microscopy.^199−202^ The characterization methods used for matrix stiffness of biomaterials or synthetic materials in matrix stiffness-related studies are summarized (Table 3).

**Table 3:** Materials (Biomaterials or Synthetic Materials) Characterized with Different Mechanical Characterization Methods to Measure a Variety of Moduli Ranging from Several Pascals to Kilopascals^a^

ref	Culture Model	Material	Stiffness Characterization Method	Measured Stiffness	Min (kPa)	Max (kPa)	Major Findings
(287)	2D Cell Culture	Polyacrylamide (PA)	AFM	Elastic Modulus	7	55	miR-29b downstream helps to maintain stem cell-like ability on different substrate stiffness’ which also causes increasing Dox resistance.
(275)	Organoid	Polyacrylamide (PA)	AFM	Elastic Modulus	0.14	5	Soft matrix promotes treatment resistance by activating NF-κB, stiff ECM enhances sensitivity to therapy through JNK signaling, both impacting apoptosis induction
(316)	2D Cell Culture	Polyacrylamide (PA)	AFM	Elastic Modulus	10	57	Soft matrix inducing autophagy and apoptosis through ROS accumulation and JNK phosphorylation
(312)	2D Cell Culture	Polyacrylamide (PA)	AFM	Storage modulus	10	57	Matrix stiffness induces ILK-mediated YAP activation-based drug resistance to Dox
(309)	2D Cell Culture	PA/Collagen I	Commercial product. Stiffness reported by manufacturer.	Elastic Modulus	0.2	50	Increasing matrix stiffness induces AMPK-driven autophagy through FAK in fibroblasts
(244)	Spheroid	Agarose	Compression	Storage modulus	1.4	30	Substrate stiffness affects spheroid formation.
(314)	Xenograft	PEG-HA	Compression	Storage modulus	0.04	1.3	Patient-derived glioblastoma cells’ MMP expression level can change with matrix stiffness and show higher resistance in stiff matrix to TMZ
(298)	3D Cell Culture	Alginate/Gelatin	Compression	Elastic Modulus	2	10	Matrix stiffness increases epithelial and mesenchymal cancer stem cell marker expressions
(278)	Xenograft	PEG	Compression	Storage modulus	2	20	Matrix stiffness directly relates to drug resistance in glioblastoma xenografts to TMZ
(301)	Spheroid	Aldehyde Sodium Alginate	Compression	Elastic Modulus	7.7	72.2	Increasing matrix stiffness correlates with CSC phenotype through YAP activation
(266)	Spheroid	Tailored GHAM Hydrogel	Magneto Rheology	Storage modulus	0.56	2.64	Matrix stiffness induces both EMT and MET based on the stiffness
(313)	3D Cell Culture	Collagen/Chitosan	Micro Strenght Testing	Storage modulus	60	290	NSCLC cells change their metabolic activity and increase drug resistance in changing stiff substrate via hyperactivation of mTOR
(274)	Organoid	Hyaluronan/Collagen I	Not reported	Shear Modulus	0.05	0.2	A coculture system of PDO and CAF is established.
(235)	Organoid	Decellularized ECM	Rheometry	Loss modulus	39	42	Cell-microenvironment mimicry done by decellularized ECM which used for the 3D printing of large tumoroids
(229)	2D Cell Culture	Polyacrylamide (PA)	Rheometry	Storage modulus	0.2	20	Changing substrate stiffness with functionalized with laminin motif peptide directly effects neurogenesis in vitro
(315)	2D Cell Culture	Polyacrylamide (PA)	Rheometry	Shear Modulus	0.1	100	Decreasing matrix stiffness promotes drug resistance to tamoxifen via the upregulation of autophagy
(276)	Spheroid	PEG	Rheometry	Storage modulus	1	7	Changing matrix stiffness on U87 cell spheroids does not significantly affects viability over Temozolomide
(135)	3D Co Culture	Collagen I/Matrigel	Rheometry	Storage modulus	1	3	Matrix stiffness induces CAF’s hypersecretion of chemoresistance-promoting exosomes of PDAC

a The culture model and reported major findings show that material based TME mimicry, biomaterial-cell interactions linked with drug resistance.

### Mimicking Natural ECM and TME

There are various approaches to mimic the native ECM and TME in terms of its composition, shape, and mechanobiological aspects. The first approach uses naturally derived materials such as collagen, alginate, gelatin, chitosan, and hyaluronic acid.^44,203−209^ With those materials, the primary approach is to mimic the ECM components and to design studies with more minor scales or using similar polysaccharides to the ECM components to screen the behavior of the cells and 3D cell clusters (spheroid, organoids, tumoroids).^210−213^ Composite structures can combine one or multiple naturally derived materials to mimic ECM construction. These approaches target the cells’ adhesiveness or 3D cell clusters into the designed mesh. One of the drawbacks of these natural materials is the batch-to-batch variations. These variations are the limitations for reproducibility and scaling up of the studies. To overcome these problems, another approach, namely fully synthetic materials, is used to mimic the ECM. This approach is based on mainly using bioinert and biocompatibility polymers such as polyethylene glycol (PEG),^214^ polycaprolactone (PCL),^215,216^ poly(vinyl alcohol) (PVA),^217^ and polyacrylamide (PA)^218,219^ with various functionalization techniques and functional groups.^220−224^ The main advantage of this approach is the controllability of the composition. It has very low batch to batch variations due to high yield bioconjugation techniques.

Controlling the structure is another critical issue with synthetic polymers, especially for mimicking tissue. For example, spatiotemporal control of synthetic polymers can result in villus-like structures.^225^ The main drawback of this approach is, in some applications, the functionalization of synthetic polymers with peptide motifs (RGD, IKVAV, etc.) for cell adhesions and transducing primary survival signaling pathways.^226−229^ To overcome these problems, hybrid-type hydrogel systems can be used. This can be achieved by modifying the polymer with various peptide motifs or creating composite hydrogels with synthetic and natural biomaterials.^212,224,230,231^

Another approach is decellularization of the actual ECM or TME from tumor tissue.^232−234^ The decellularization can provide the tissue ECM/TME without any attached cells. This approach is useful, especially when working the similar conditions, such as culturing breast tumoroids in decellularized breast cancer TME.^235,236^ The Matrigel, a gold standard of the 3D cell culture systems, is based on decellularized Engelbreth–Holm–Swarm (EHS) mouse sarcoma tissue.^237^ The main drawback of these systems is the batch-to-batch variations and low reproducibility in experiments.^230,238,239^ Two main approaches use hydrogels as supportive hydrogel systems to embed the cells into hydrogel systems. To do that, most of the time, several biological molecules should be implemented in the hydrogels so they can support the survival and proliferation of the cells. One of the main biological molecules used for hydrogels is small peptide sequences. The most commonly used one is fibronectin derived RGD peptides. RGD peptides are arginine-glycine-aspartic acid-based motifs, and they were discovered in the 1980s as a cell adhesion motif in fibronectin.^240,241^ Without cell adhesion motifs, cells are not attached to the hydrogel systems and here will be referred to as nonadhesive hydrogel systems. These systems are primarily used for the formation of 3D cell clusters due to their nonadhesive features. Also, cells can be seeded over the hydrogel as a coating. Those hydrogels are prepolymerized, and petri dishes or similar cell culture growth plates are coated with the prepolymerized solutions. After the coating, cells either attach to the surface of the polymer by adhesive molecules or do not attach and act as nonadhesive.^242,243^ Of note, the nonadhesiveness is primarily helpful in generation of 3D cell clusters.^244^ Collectively, hydrogel systems incorporated with various techniques can be implemented to understand mechanobiological processes in relation to tumor biology.

## Matrix Stiffness as a Mechanical Modulator of Tumor Biology and Chemoresistance

### Matrix Stiffness Regulates EMT and Chemoresistance

Epithelial-mesenchymal transition (EMT) is a process where epithelial cells lose their apical-basal polarity in conjunction with the cell–cell adhesiveness via decreased expression of E-cadherin protein.^245^ With the decrease in E-cadherin expression, the EMT program starts to be activated, and epithelial cells begin to gain mesenchymal phenotype by reorganizing their cytoskeleton, especially the actin stress fibers^246^ mediating the epithelial cells to change their phenotype toward more elongated shape for high invasiveness.^247−250^ The TGF-β pathway is crucial for the EMT program observed in carcinomas with forming SMAD complexes. The high elastic modulus of a surface may help induce EMT through the activation of TGF-β signaling pathway. Integrin α_v_ works as an intermediate transducer between fibronectin and TGF-β1 complex to promote stiffness-induced and TGF-β-based EMT. The involvement and the activation of these signaling pathways have also been reported to play a role in drug resistance in cancers including breast, colon, pancreatic, and ovarian, where oxaliplatin and cisplatin-based drugs are used frequently. The SNAIL and SLUG have been reported to play a key role, especially in the tissue remodeling and drug resistance of oxaliplatin and cisplatin-based drugs.^251,252^

Additionally, integrin α_v_ induces the production of LOX enzymes and supports the stiffness via cross-linking of collagen fibers. In a study conducted by Fan et al., polyacrylamide hydrogels with tunable stiffness were used to investigate proliferation, phenotypic switching, and chemoresistance of ovarian cancer cells. 0.5, 4, and 25 kPa polyacrylamide gels were prepared, and it was reported that stiffness induced the matrix-induced YAP translocation and proliferation. In contrast, low stiff substrates induced EMT through increasing mesenchymal markers such as vimentin and decreasing epithelial markers such as E-cadherin and β-catenin expression. Additionally, low stiffness matrices could induce chemoresistance in ovarian cancer cells through the upregulation of ABCB1 and ABCB4 platinum drug resistance genes.^253^

Wnt signaling pathway is also essential in EMT program initiation. The translocation of β-catenin will promote the expression of ZEB1, TWIST, and SLUG, and the direct interaction of β-catenin with SNAIL will provide a synergistic effect for the transcriptional function of β-catenin. Xu et al. showed that the stiffness of the matrix could activate the NEAT1-Wnt/β-Catenin pathway and induce EMT and proliferation as well as drug resistance to doxorubicin in liver cancer using HepG2 cells and micropillar PDMS based elastomer.^254^

**Figure 3:**
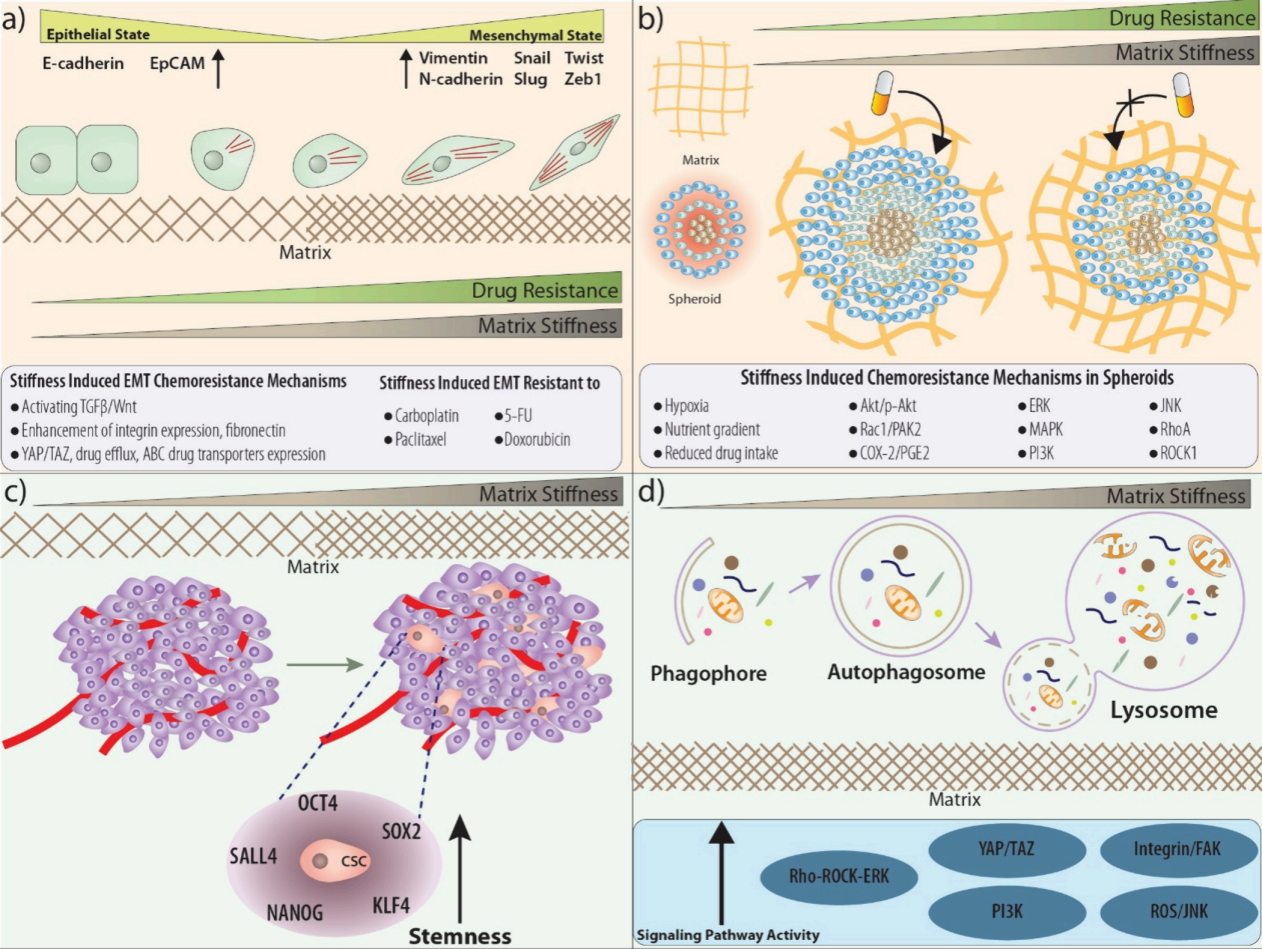
Matrix stiffness may be related to the EMT and its reverse form MET with related mechanisms such as activating TGFβ/Wnt, YAP/TAZ, increased integrin, and fibronectin expression levels as well as the drug efflux pumps, ABC transporters in chemoresistance (A), while spheroid formation and chemoresistance with the involvement of different mechanisms such as hypoxia, nutrient gradient, and reduced drug intake via Akt/p-Akt, Rac1/PAK2, COX-2/PGE2, PI3K, ERK, MAPK, JNK, RhoA, ROCK1 signaling pathways (B), also, cancer stemness in relation to matrix stiffness may be increased through regulating the levels of CSC-related proteins such as SALL4, OCT4, SOX2, KLF4 and NANOG (C). Also, a number of signaling pathways such as Rho-ROCK-ERK, YAP/YAZ, Integrin/FAK, ROS/JNK and PI3K involved in regulating matrix stiffness may induce macroautophagy and cell death (D).

The NOTCH signaling is one of the primary pathways activating the EMT program in lung, breast, and pancreatic carcinomas. The NOTCH signaling is known to induce EMT via the expression of vimentin, fibronectin, and transcriptional regulation of the SNAIL and SLUG. Also, the NOTCH signaling pathway can work alongside the TGF-β to induce the EMT program.^257,258^ The EMT and its relationship to matrix stiffness and drug resistance are summarized (Figure 3a).

Various mitogenic growth factor receptors can synergically work with p38 MAPK, ERK-MAPK, PI3K/AKT, and JNK pathways and are closely associated with inducing EMT program, proliferation, migration, and cell growth.^259,260^ Additionally, epidermal growth factor (EGF) activates EMT through MEK-ERK and STAT3 pathways and downregulates the E-cadherin expression, promoting TWIST and N-cadherin, and vimentin. EGF can also induce an EMT program by a crosstalk with other signaling pathways such as TGF-β. Hepatocyte growth factor (HGF) induces the expression of SNAIL to activate EMT, invasion, and eventually tumor metastasis. Fibroblast growth factor (FGF) is also related to activating MAPK and MEK-ERK pathways which are known for inducing the EMT program. In the study conducted by Jingyuan et al., they investigated the effect of matrix stiffness on oral squamous cell carcinoma dormancy. They analyzed 127 patients for stiffness-related mechanical stress on tumor behaviors. They found that stiff matrix can cause poor survival, repopulating of tumors, as well as increasing drug resistance and invasiveness based on EMT induction. Also, increasing matrix stiffness can cause DNA damage and activate the cyclic GMP-AMP synthase-stimulator of interferon genes (cGAS-STING) signaling.^261^

Fibronectin is linked with matrix stiffness in the EMT program via stretching Fibronectin type III through additional growth factors and ECM binding sites. Collagen also works in parallel with fibronectin to increase the tension. Vimentin is assembled into intermediate filaments and is closely related to the mechanotransduction of various signaling pathways including ERK and ROCK.^262−265^ Fibrillar matrix conversion downregulates epithelial markers while upregulating the mesenchymal factors. The fibrillar matrix stiffens to promote EMT via microtubule-based force generation. It acts as a positive feedback loop that stiffens the matrix, promotes growth factor binding and matrix deposition in fibrillogenesis, and stiffens the matrix more. The matrix stiffness also promotes EMT by inducing transcription factors such as TWIST. Shou et al. have created a magnetic hydrogel that can be controlled wirelessly. The hydrogel has a dynamic 3D structure with adjustable stiffness, achieved by using gelatin, hyaluronic acid, RGD motifs, and thiolated magnetic microparticles. The stiffness range of the end product is from 0.5 to 2.7 kPa, and it was used to grow breast cancer cell lines in a spheroid shape. The study found that a stiffer matrix could increase tumor malignancy and hypoxia, leading to EMT. However, the researchers also discovered that softening the stiffened matrix could reverse EMT and promote MET. Interestingly, when spheroids were treated with chemotherapeutics like doxorubicin, the antitumor effect of the drug was reduced in stiffer matrices.^266^

The drug resistance mechanisms can be seen in various cancer types such as colorectal and lung cancers. Even though the mechanisms are not clearly understood, some of the drug resistance mechanisms related to TME and its stiffness have been reported. For example, TGF-β1 and hyaluronan are essential ECM components in the drug resistance mechanism induced by the EMT program. IL-6 is also related to TGF-β1 and EMT, which is linked to cisplatin drug resistance in lung cancer, while gemcitabine resistance is linked to IL6 family protein oncostatin M and hypoxia. Additionally, upregulation of EMT markers can promote cancer cells to escape immune cells, especially working parallel with PDL-1 to resist nivolumab—an immunotherapeutic agent.

### Matrix Stiffness Regulates the Growth of Tumor Spheroids, Tumoroids, and Chemoresistance

Spheroids are three-dimensional tumor cell aggregates that will favor the cell–cell and cell–environment interactions.^267^ The shaping pattern of spheroids in vitro is essential for mimicking tumorigenesis and differentiation in cancer. Since they have three-dimensional shapes, they have different layers for various types of cells to mimic solid tumors.^268^ Necrotic cells will be in the very inner layer while migrating and proliferating cells are in the outermost layer, and the nondividing quiescent cells lay in the middle of these layers.^269^ Tumor spheroids cancer research and are used for invasion and migration processes mimicking the tumor progression.^270^ Tumor spheroids can be generated using in vitro techniques such as magnetic levitation, microculture plates, hanging drop, 3D printing, and natural, synthetic, and hybrid hydrogels.^271^ Drug screening and resistance applications are the most advanced use of tumor spheroids. Their application range is much bigger than two-dimensional cell line studies. In three-dimensional studies, the microenvironment’s various chemical and mechanical changes will affect the drug resistance.^272^ How the spheroid formation is related to matrix stiffness is shown (Figure 3b).^273^ Moreover, different drug resistance mechanisms related to cancer stem cells, cell–cell and cell–ECM interactions can also be assessed with the presence of the microenvironment and the tumor spheroids. In a study by Luo et al., a hyaluronan-gelatin composite hydrogel system with PEG-DA was used to investigate potential patient-derived organoid coculture systems with CAFs. PDOs were treated with capecitabine and 5-FU, as well as oxaliplatin and irinotecan for 120h. As a result, increased drug resistance in colorectal cancer cells in a crosstalk with CAFs was reported.^274^ In another study, circulating tumor cells forming the spheroid shapes, they exhibited more drug resistant phenotypes due to the physical barrier did not allow the drug intake to the core of the spheroids.^273^ In a study by Drain et al., different models of triple-negative breast cancer, such as organoids, xenografts, and spheroids, exhibited varying levels of resistance to chemotherapy depending on the stiffness of the matrix. To further investigate this observation, they utilized polyacrylamide gels modified with basement membrane components and had adjustable stiffness ranging from 0.14 to 5 kPa. The researchers reported that a matrix with low stiffness could promote treatment resistance by activating NF-kB and JNK signaling, which impedes apoptosis induction. Conversely, a stiff matrix enhances proapoptotic JNK activity and affects chemoresistance to paclitaxel.^275^ Furtermore, Bruns et al. conducted a study on PEG-based hydrogels with stiffness ranging from 1 to 7 kPa, including a dual stiff model. They aimed to investigated growth, invasion, proliferation of glioblastome spheroids and performed a drug screening. They utilized 4-arm PEG-acrylamide functionalized with RGD peptide sequence and cross-linked with enzymatically degradable peptide (VPM). Interestingly they reported no significant differences in Temozolomide treatment response between soft and stiff scaffolds.^276^ Li et al. conducted a study using a collagen-alginate hydrogel system to grow estrogen receptor-positive breast cancer spheroids and observed their response to varying hydrogel stiffness. The hydrogels were prepared with stiffness ranging from 0.0469 to 0.902 kPa and were used for spheroid formation and growth for 16 days. The study found that spheroids grew larger in lower stiffness hydrogels than higher stiffness hydrogels. Additionally, the study measured Doxorubicin IC50 values for spheroids on the 7^th^ and 16^th^ day with limiting stiffness values and reported that spheroids placed in softer hydrogels showed 1.8-fold greater chemoresistance compared to those in stiffer hydrogels.^277^ Another critical study conducted by Wang and colleagues explored using a hydrogel system with varied stiffness for glioblastoma xenografts in a 3D tumor environment. They functionalized an 8-arm PEG norborene using a cross-linker with MMP cleavable peptide and linear PEG-SH. Hydrogels were prepared with stiffness levels ranging from 0.04 to 26.6 kPa, and it was reported that lower stiffness levels led to cell proliferation, while higher stiffness levels induced chemoresistance to Temozolomide, and the expression of RhoA and ROCK1 were upregulated. The study further reported that cell viability increased by over 60% as stiffness levels increased from 0.04 to 26.6 kPa.^278^

### Matrix Stiffness Regulates Stemness and Chemoresistance

Cancer stem cells (CSCs) have unique phenotypes like normal stem cells, and they have an ability to self-renewal for the formation of new tumors. Within tumor mass, CSCs have been reported as one of the drivers of chemoresistance, and this process is often linked with EMT.^136^ One of the CSC markers includes a transmembrane protein CD44 which is involved in ECM-cytoskeleton signaling. Further, a subtype of CD44 called CD44v mediates the metastasis process and stemness characteristics.^279^ Another essential protein is the integrin α6 subunit known to mediate the tumor sphere formation and taxane resistance.^280,281^ Moreover, the prominin-1 (CD133) facilitates cancer stem cell self-renewal. The overexpression of prominin-1 is linked to chemoresistance, especially in platinum-based ones, such as paclitaxel and cisplatin.^282,283^ Chemoresistance in nonsmall cell lung cancer is partly regulated by CD44 and EpCAM complex.^279^ CSCs broadly express the aldehyde dehydrogenase (ALDH1) and mediate chemoresistance by regulating cell-cycle checkpoints and nucleic acid repair pathways. Further, ALDH1 is also known to be involved in the detoxification of drug-mediated aldehydes in cancer cells and hence promoting chemoresistance. In addition, the motility-related protein-1 (MRP-1/CD9) and CD24 exhibit therapeutic resistance in CSCs.^284,285^ The influence of matrix stiffness on cancer stemness has been studied in a study by Tan et al. Polyacrylamide gel with various stiffness starting at 2 to 20 kPa was combined with the human HCT116 cancer cell line. And they seeded cells on collagen coated PA gels. They have reported that stem markers, like CD133, ALDH1, and Lgr 5, are induced by matrix stiffness. Also, dephosphorylation of YAP and integrin-β1/FAK pathway induce stemness phenotype as well.^286^ In a publication by Li et al., an investigation of ECM stiffness for stem cell-like abilities of osteosarcoma cells showed that microRNA-29b signaling is an essential factor for stem cell-like ability increasing with the low stiff matrix. They used collagen type I coated polyacrylamide gel with a range of 7 kPa to 55 kPa stiffness and reported that low stiff matrix induces miR-29 downregulation and activates the PI3K/Akt and Stat3 signaling. They also reported that softer substrates enhance the stem-cell-like characteristics and cause increasing drug resistance to doxorubicin. They showed a correlation between the stemness markers and increasing levels of IC50 values against doxorubicin.^287^

Both canonical and noncanonical Wnt signaling pathways play a significant role in promoting cancer stem cell phenotypes. Previous reports demonstrated the involvement of the Wnt signaling pathway in gaining stem cell characteristics and chemoresistance in colon cancer.^288,289^ Furthermore, the Notch pathway plays a critical role in the self-renewal ability of cancer stem cells, the EMT, and in the chemoresistance of platinum-based chemotherapeutics.^290,291^ Lastly, both the Hedgehog and JAK/STAT pathways are reported to mediate the self-renewal capacities of cancer stem cells and chemoresistance in various cancer types.^292−295^ Verteporfin, an FDA approved YAP/TAZ inhibitor, has been reported to suppress cancer stem cell phenotype and progrestin resistance in mesothelioma and endometrial carcinoma.^296^ In addition, phase I/II clinical trial for treating EGFR-mutated glioblastoma patients with verteporfin has been initiated.^297^ The matrix stiffness relationship with stemness in cancer is summarized (Figure 3c).

Shah et al. recently conducted a study exploring the impact of stiffness on breast cancer cell stemness. To do so, they created alginate-gelatin composite hydrogels that ranged in stiffness from 2 to 10 kPa. These hydrogels were then used to encapsulate MDA-MB-231 and MCF-7 breast cancer cell lines, which were perfused to mimic physiological fluid flow. Over 14 days, the researchers observed that cells tended to aggregate more in softer gels. Moreover, they discovered that cancer stem cell populations (both epithelial and mesenchymal) increased as the matrix stiffness and pH levels became more acidic.^298^ In the research conducted by Li et al., they used 3D collagen, fibrinogen and Matrigel to investigate mechanical forces that are related to cancer cell stemness in breast cancer. They prepared gel systems with stiffness ranging from 0.045 to 0.45 kPa and seeded breast cancer cells. The results showed that low stiff matrices activate integrin β1/3 receptors and cytoskeleton/AIRE axis due to stem-like phenotypes with upregulation of breast cancer stem cell marker ALDH1+. While beyond kPa level, stiffness of the matrices can cause apoptosis and structural damage.^299^ The study done by Liu et al. showed that in breast cancer, the stiffness of the matrix is highly associated with drug resistance to chemotherapeutics and regulates CSC enrichment via TAZ-NANOG phase separation. They used breast cancer cell lines on a polyacrylamide gel system ranging from 0.5 to 9 kPa. Docetaxel and cisplatin treatment showed that stiff matrix significantly lowers apoptosis than softer ones. Also, in chemoresistant groups, breast cancer samples showed higher ALDH1+CK+ CSCs. They also reported that TAZ upregulation in stiff matrices showed upregulation of SOX2 and OCT4, stemness related TFs, then softer matrices. Another study reported that NANOG mediates SOX2 and OCT4 TFs to induce differentiation of stem cells.^300^ Also Li et.al. showed that increased matrix stiffness correlates with increased levels of liver cancer stem cells. They used an aldehyde sodium alginate (ASA) hydrogel system with a stiffness range from 7.7 to 72.2 kPa and reported that YAP signaling might mediate stemness in liver cancer.^301^

### Matrix Stiffness Regulates Autophagy and Cell Death

Autophagy plays a critical role in orchestrating protein accumulation, immunological response, and various disorders ranging from cardiovascular to neurodegenerative diseases, and cancer.^302^ There are several steps for autophagy, the first one is its initiation. The initiation can be induced by several factors such as stress factors (ranging from cellular to organelle level), infection, hypoxia, inflammation mediated by JNK, p53, CD46, CD40, and several other signaling cascades. The autophagy is activated by various stimuli including ULK1 (Unc-51 Like Autophagy Activating Kinase 1) complex formation and PI3KC3 (Phosphatidylinositol 3-kinase catalytic subunit type 3) complex phosphorylation.^8,302^ Also, in tumor and TME crosstalk, studies showed that cardiotropin-1, CTF1, is one of the mediators and highly correlating with activating autophagy and regulating migration, invasion, and metastasis in cancers.^303^ Finally, the closure occurs with the fusion enhanced by SNARE and HOPS.^304^ Then the enclosed autophagosomes interact with lysosomes and degrade the dysfunctional components in the autophagosomes.^305^ The microenvironment plays an essential role in autophagy. Since the loss of tissue homeostasis is crucial for malignancy, the ECM components related to stress are also directly associated with autophagy. Various stress types can affect the activation of autophagy; for example, fluid stress around 0.05–1.2 Pa level will activate the autophagy in different carcinomas, while several pascals of shear stress can result in the cell death. Shear stress is highly related to cytoskeleton regulation. The stress level can increase due to the increasing level of cross-linking between collagen fibers and other ECM components. The stiffer the matrix gets, the more changes in cell–ECM interaction will be altered including the expression of focal adhesions, cell–cell junctions, and integrins.^306−308^ Furthermore, the matrix stiffness regulates the Hippo-YAP/TAZ signaling, which is directly related to autophagy. Inhibiting this pathway can decrease autophagy as well as drug resistance. JAK inhibition is also linked to autophagy; inhibiting JAK will induce autophagy^309−311^ (Figure 3d).

Recent research undertaken by Qin et al. utilized polyacrylamide hydrogels with varying degrees of stiffness to assess the impact of matrix stiffness on the induction of drug resistance in breast cancer cells. By employing 10, 38, and 57 kPa stiff polyacrylamide gels, they discovered that 38 kPa gels induced doxorubicin drug resistance in breast cancer cells through ILK-mediated YAP activation.^312^ In another study, Fu et al. revealed that nonsmall cell lung cancer cells grown in 3D collagen-chitosan composite hydrogel scaffolds exhibited higher drug resistance than those grown in 2D culture due to hyperactivation of mTOR. They created scaffolds ranging from 60 to 290 kPa and treated them with cisplatin-based drugs.^313^ In another study by Zhu et al., an 8-arm PEG norbornene was utilized and functionalized with RGD peptide, dithiol PEG, and thiolated hyaluronic acid. The team then cross-linked this hydrogel system with an MMP cleavable peptide sequence. The resulting stiff gel had a highly tunable range of 0.04 kPa to 1.3 kPa and was polymerized via UV light. In another study, glioblastoma multiforme patient-derived xenografts were cultured for 21 days and treated with Temozolomide. They discovered that MMP expression was higher in less stiff regions, while increased stiffness led to greater drug resistance.^314^ In a publication by Anlaş et al., breast cancer cells exhibited an increased autophagy in soft matrices and became more resistant to tamoxifen, an estrogen receptor modulator. They used polyacrylamide gels ranging from 0.1 to 100 kPa, cultured breast cancer cell lines in matrices, and treated with tamoxifen. They showed that soft matrices induced chemoresistance correlating with upregulation of autophagy while inhibiting autophagy on soft matrices decreased the chemoresistance in breast cancer cells.^315^ In a study conducted by Chen et al., polyacrylamide gels with stiffness range from 10 to 57 kPa showed that breast cancer cell line MDA-MB-231 grown in the low stiff matrices upregulated autophagy with the activation of ROS/JNK signaling pathway.^316^ In a study by Hupfer et al., collagen type-1 coated hydrogels with various stiffness ranging from 0.2 to 50 kPa were used. Their results showed that AMPK levels were elevated in stiffer conditions while mTOR levels were unaffected in fibroblasts. They also showed that the AMPK based changes were closely related to integrin alphaV-FAK signaling pathway and dependent on ITGAV.^309^ The major findings of matrix stiffness related studies discussed in several stiffness related chemoresistance studies are summarized (Table 3).

## Conclusions and Future Perspectives

Certain approaches exploiting mechanotransduction could be utilized in reverting drug resistance and hence employing matrix stiffness in favor of patients. Matrix stiffness is linked to drug resistance via the induction of EMT, autophagy, proliferation, and cancer stemness in cancer cells. Through these alterations, cancer cells can develop resistance to different types of drugs and their combinations. Importantly, some of these changes can be reversible, especially via the enzymes secreted by cancer cells cleaving the ECM components and hence decreasing the stiffness. Matrix stiffness and its effect on mechanotransduction of signaling pathways are emerging research areas. Excellent reviews in this field have so far provided preclinical and clinical therapy intervention strategies.^114,120,317^ Understanding the underlying mechanisms of these processes will be instrumental in tailoring novel therapeutic approaches in cancer with an ultimate aim to revert matrix stiffness and hence the drug resistance.

Future investigations in relation to understanding the effects mechanotransduction in drug resistance mechanisms will be instrumental in conjunction with using clinically relevant model systems such as Patient-Derived Organoids (PDOs). PDOs have been successful in mimicking patient tumors’ drug response and their genetic/phenotypic heterogeneity. Therefore, altering the mechanotransduction properties of supporting matrix to grow PDOs ex vivo might be ideal to recapitulate personalized drug response for patients. Expanding this approach toward incorporating stromal cells into PDO culture system might help in measuring the drug response in cancer cells while considering the heterotypic interactions between cancerous and noncancerous cells including immune cells, cancer-associated fibroblasts and endothelial cells. Collectively, more sophisticated model systems to study mechanotransduction in cancer drug resistance developed would pave the way to advanced personalized medicine by tailoring more effective interventions to overcome or control drug resistance in cancer.
